# Real-World Effectiveness and Safety of Eliglustat in Adult Patients with Gaucher Disease Type 1: A Multicenter Retrospective Study in China

**DOI:** 10.3390/jcm15062323

**Published:** 2026-03-18

**Authors:** Yongxin Zhou, Zijian Hao, Qilin Zhuang, Bing Han

**Affiliations:** 1 Department of Hematology, Peking Union Medical College Hospital, Chinese Academy of Medical Sciences & Peking Union Medical College, Beijing 100730, China; zhouyong21@mails.tsinghua.edu.cn (Y.Z.); hao-zijian@student.pumc.edu.cn (Z.H.); 202101067@student.pumc.edu.cn (Q.Z.); 2 Eight-Year Medical Doctor Program, Chinese Academy of Medical Sciences & Peking Union Medical College, Beijing 100730, China

**Keywords:** Gaucher disease type 1, eliglustat, substrate reduction therapy, real-world data

## Abstract

**Background/Objectives:** Eliglustat is an oral therapy for Gaucher disease type 1 (GD1) that may reduce infusion-related logistical burden, particularly in resource-constrained settings. Post-approval evidence from routine clinical practice in China remains limited. This study evaluated its real-world effectiveness and safety in Chinese adults with GD1. **Methods:** This retrospective, multicenter study included adults with GD1 receiving eliglustat monotherapy for ≥6 months. Outcomes included plasma glucosylsphingosine (lyso-Gb1), hemoglobin (HGB), platelet count (PLT), liver and spleen volumes, and adverse events (AEs). Depending on distribution, paired changes were analyzed using paired *t* tests or Wilcoxon signed-rank tests. *p* < 0.05 was considered statistically significant. **Results:** Nineteen patients were included in the effectiveness analysis, with a median follow-up of 7 months (range, 6–9). Lyso-Gb1 decreased from 468 to 210 ng/mL (*p* < 0.0001). HGB increased from 123 to 131 g/L (*p* = 0.147); among six patients with baseline anemia, 83.3% improved and 33.3% normalized. PLT increased from 109 to 132 × 10^9^/L (*p* = 0.019); among 12 patients with baseline thrombocytopenia, 58.3% improved. Liver volume decreased from 1808 to 1747 mL (*p* = 0.016) (1.22 to 1.01 multiples of normal; *p* < 0.001). Spleen volume decreased from 473 to 452 mL (*p* = 0.016) (4.69 to 5.17 multiples of normal; *p* = 0.015). Lyso-Gb1 reduction was greater in patients without prior enzyme replacement therapy (ERT) exposure than in those with prior ERT exposure (−55.1% vs. −43.1%; *p* = 0.049). In the safety analysis group (*n* = 90), suspected drug-related AEs occurred in 27.8% of patients, mainly gastrointestinal or skin-related, and were limited to grade I/II. No serious AE or treatment discontinuation occurred. **Conclusions:** In routine clinical practice in China, eliglustat was associated with rapid substantial reductions in plasma lyso-Gb1, early improvements in hematologic and visceral parameters, and favorable short-term tolerability in adults with GD1.

## 1. Introduction

Gaucher disease (GD) is a rare autosomal recessive lysosomal storage disorder, with a reported prevalence of 0.7–1.75 per 100,000 worldwide [1] and an estimated prevalence of 0.15–0.22 per 100,000 in China [2,3]. GD is caused by pathogenic variants in *GBA1* (glucosylceramidase beta 1), which encodes lysosomal β-glucocerebrosidase (GCase) [4]. Deficient GCase activity impairs lysosomal degradation of glucosylceramide (GluCer) and related glycosphingolipids, resulting in lipid accumulation in macrophages and the formation of characteristic Gaucher cells. These storage macrophages contribute to chronic immune and inflammatory dysregulation and drive progressive multi-organ involvement [5,6,7]. Clinically, GD shows a broad and heterogeneous spectrum, including hepatosplenomegaly, anemia, thrombocytopenia, and skeletal complications such as osteoporosis and bone crises; some patients also develop neurological involvement [8]. Based on the presence and course of neurological symptoms, GD is classified as non-neuronopathic (GD1), acute neuronopathic (GD2), and chronic neuronopathic (GD3). Among available biomarkers, glucosylsphingosine (lyso-Gb1) closely reflects substrate burden and is widely used for diagnosis, disease monitoring, and evaluation of treatment response [9,10].

Current disease-specific management for GD includes enzyme replacement therapy (ERT) and substrate reduction therapy (SRT). ERT restores deficient enzyme activity through biweekly intravenous infusions of recombinant GCase (e.g., imiglucerase, velaglucerase alfa) [11]. However, long-term ERT requires frequent hospital visits and sustained adherence, and imposes practical burdens related to infusion scheduling and cold-chain storage. In routine practice, these barriers may disrupt continuity of care, particularly in resource-constrained settings, underscoring the need for an effective oral alternative [12,13]. SRT reduces substrate accumulation by inhibiting GluCer synthesis upstream. Miglustat, the first approved SRT, has had limited use in practice because of modest effectiveness and significant adverse effects (AEs) [14]. Eliglustat is a next-generation SRT that selectively inhibits glucosylceramide synthase (GCS), a key enzyme in GluCer biosynthesis [15]. Because eliglustat exposure depends on cytochrome P450 2D6 (CYP2D6), eligibility and dosing are guided by metabolizer status, including ultrarapid (URMs), extensive (EMs), intermediate (IMs), or poor metabolizers (PMs) [16]. Importantly, both ERT and eliglustat have limited ability to cross the blood–brain barrier and therefore do not adequately address neurological manifestations in neuronopathic GD (GD2/GD3), consistent with their primary role in systemic disease management [17].

Clinical trials and real-world studies have shown that eliglustat achieves efficacy comparable to ERT, with improvements in hematologic, visceral, and skeletal outcomes in both patients without prior treatment and those who switch from ERT [18,19,20]. Follow-up data of up to eight years support sustained effectiveness and a stable safety profile [21], and several clinical guidelines recognize eliglustat as a first-line option for eligible adults with GD1 [16,22]. In October 2022, eliglustat tartrate capsules (Beijing Chiral-Tech Pharmaceutical Co., Ltd., Beijing, China) were approved by the China National Medical Products Administration for long-term treatment of adult patients with GD1 who have CYP2D6 EM, IM, or PM phenotypes, expanding access to an oral alternative to infusion-based care in China. Despite this regulatory milestone, post-approval evidence from routine clinical practice in China remains scarce. Most evidence on eliglustat comes from Western clinical trials and registries, whereas multicenter real-world data on its effectiveness and safety in Chinese adults with GD1 are limited. To address this gap, we conducted a multicenter retrospective study to evaluate the real-world effectiveness and safety of eliglustat in Chinese adults with GD1.

## 2. Materials and Methods

### 2.1. Study Design and Patients

**Figure 1:**
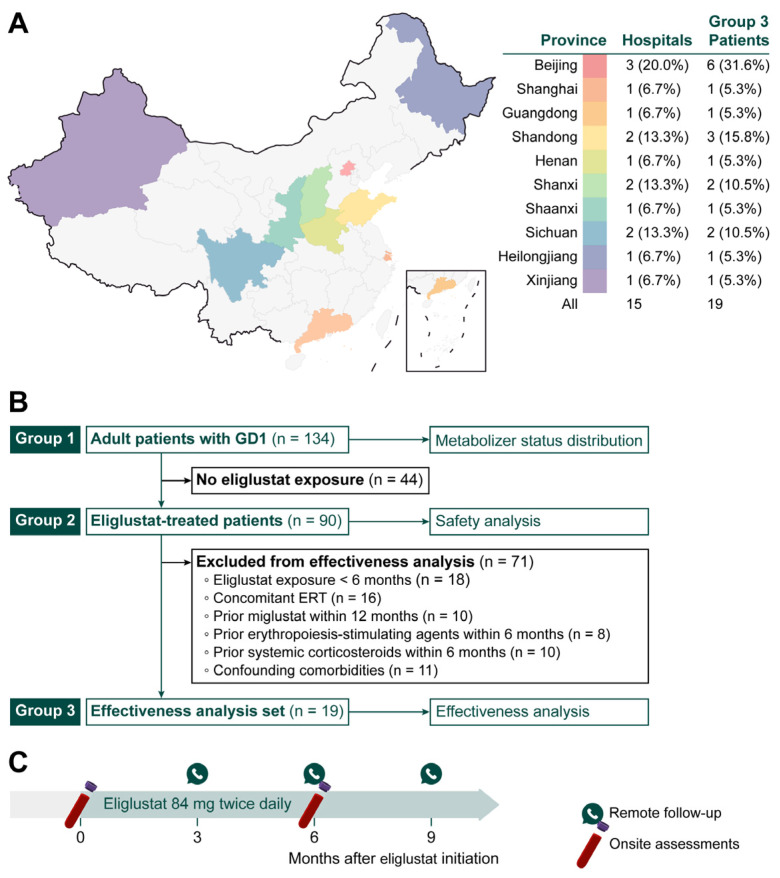
Study design. (**A**) Geographic distribution of the 15 participating hospitals across China and the number of sites and patients included in the effectiveness analysis set (Group 3) by province. (**B**) Flow diagram of the three nested study groups. (**C**) Overview of treatment and assessment timeline. Abbreviations: GD1, Gaucher disease type 1; ERT, enzyme replacement therapy.

This retrospective, multicenter study included adult patients with GD1 from 15 hospitals in China between November 2023 and July 2024. Participating centers were distributed across multiple regions of China (Figure 1A). GD1 was confirmed by the identification of pathogenic *GBA1* variants together with reduced GCase activity (defined as <30% of the lower limit of normal) [23].

To address different study objectives, patients were analyzed in three nested groups (Figure 1B). Group 1 included all adult patients with GD1 and was used to describe the distribution of metabolizer status. Group 2 comprised patients in Group 1 who received eliglustat and was used for safety analyses. Group 3, a subset of Group 2, consisted of patients who met prespecified criteria for effectiveness analyses.

For effectiveness analyses, inclusion criteria included: (1) age ≥ 18 years; (2) confirmed GD1; and (3) continuous eliglustat monotherapy for at least six months. Exclusion criteria included: (1) treatment with miglustat within 12 months prior to eliglustat initiation; (2) use of erythropoiesis-stimulating agents or systemic corticosteroids within 6 months prior to eliglustat initiation; and (3) comorbidities or conditions that could confound effectiveness analysis, particularly those that could independently contribute to anemia, thrombocytopenia, hepatomegaly, or splenomegaly. These included (but were not limited to) malignancies, liver disease, hematologic disorders, pulmonary complications, and cardiac abnormalities.

Written informed consent was obtained from all participants. The study was approved by the Ethics Committee of Peking Union Medical College Hospital (Approval No.: I-25PJ1535; approval date: 11 July 2025) and was conducted in accordance with the Declaration of Helsinki.

### 2.2. Study Treatment

Eliglustat tartrate capsules (Beijing Chiral-Tech Pharmaceutical Co., Ltd., Beijing, China) were prescribed according to metabolizer status. EMs and IMs received 84 mg twice daily [24]. Dose reduction was permitted only when adverse events (AEs) impaired tolerability; in such cases, the dose could be reduced by 50% (to 84 mg once daily) at the investigator’s discretion. Concomitant medications were systematically reviewed at treatment initiation to minimize drug–drug interactions.

### 2.3. Effectiveness and Safety Assessments

Clinical data were collected at baseline and at the first follow-up visit after at least six months of eliglustat therapy for patients included in the effectiveness analysis. Baseline characteristics included demographics, disease duration, metabolizer status, prior ERT exposure, and splenectomy status. Effectiveness outcomes included lyso-Gb1, hemoglobin (HGB), platelet count (PLT), and liver and spleen volumes. Given the short follow-up, analyses focused on early changes in lyso-Gb1 and hematologic parameters, whereas normalization of hematologic abnormalities and changes in organ volumes were considered supportive and exploratory. All analyses were performed using complete-case data, without imputation for missing values. Organ volumes were assessed by magnetic resonance imaging [25], and reported as absolute values and as multiples of normal (MN), calculated using 25 mL/kg for normal liver volume and 2 mL/kg for normal spleen volume [26]. Patients with prior splenectomy or partial splenectomy were excluded from spleen-volume analyses because splenic surgery alters baseline anatomy and volume, limiting comparability and interpretability.

Safety was assessed by documenting all AEs, graded according to the Common Terminology Criteria for Adverse Events (CTCAE) version 5.0. All AE entries and grades were reviewed centrally for consistency. No additional protocol-mandated safety tests were performed. Patient-reported symptom changes were collected via online follow-up approximately every three months to supplement AE capture.

All information was primarily extracted from electronic medical records and, when necessary, supplemented or verified through direct communication with local physicians to ensure completeness and accuracy.

### 2.4. Statistical Analysis

Statistical analyses were performed using R (version 4.4.1; R Foundation for Statistical Computing, Vienna, Austria). All tests were two-sided, and *p* < 0.05 was considered statistically significant. An a priori sample size estimation for paired comparisons (α = 0.05; power = 80%) indicated that approximately 18 patients would be sufficient for the key effectiveness outcomes; the assumed effect size and variability were conservatively derived from published trial reports [18,21].

Categorical variables are summarized as frequency (percentage). Continuous variables are summarized as median (range) and assessed for normality using the Shapiro–Wilk test. For paired comparisons, paired *t* tests were used for normally distributed data and Wilcoxon signed-rank tests for non-normally distributed data. For subgroup analyses, independent-samples *t* tests or Mann–Whitney U tests were applied as appropriate; these analyses were considered exploratory with no adjustment for multiple testing.

Effect sizes were reported with 95% confidence intervals (CIs): Cohen’s d for paired and independent-samples *t* tests, and r (computed as Z/√n) for Wilcoxon signed-rank and Mann–Whitney U tests. Effect sizes were interpreted using conventional thresholds (Cohen’s d: 0.2 small, 0.5 medium, 0.8 large; r: 0.1 small, 0.3 medium, 0.5 large). Percentage change was calculated as (follow-up − baseline)/baseline × 100% for each patient.

## 3. Results

### 3.1. Baseline Characteristics

CYP2D6 genotyping was available for 134 Chinese adults with GD1, identifying 78 (58.2%) EMs and 56 (41.8%) IMs, with no URMs or PMs detected. Among patients who initiated eliglustat and were included in the safety analyses (*n* = 90), 55 (61.1%) were EMs and 35 (38.9%) were IMs. Among those meeting the prespecified criteria for the effectiveness analysis (*n* = 19), 16 (84.2%) were EMs and 3 (15.8%) were IMs.

The effectiveness analysis group included 9 males and 10 females, with a median age of 36 years (range, 18–55) at eliglustat initiation and a median age of 25 years (range, 2–44) at diagnosis. Eight patients (42.1%) had undergone splenectomy (6 total and 2 partial). Eight patients (42.1%) had received prior ERT for a median duration of 10 months (range, 1–15) and transitioned to eliglustat due to suboptimal response or waning effectiveness; the median interval between ERT discontinuation and eliglustat initiation was 3 months (range, 3–5). No patient had documented current tobacco use or heavy alcohol intake. Concomitant medications were reviewed at eliglustat initiation, with particular attention to agents with potential inhibitory or inducing effects on CYP2D6 or cytochrome P450 3A (CYP3A). Baseline demographic and clinical characteristics are summarized in Table 1.

### 3.2. Effectiveness Analysis

Over a median follow-up of 7 months (range, 6–9), body weight did not change significantly. Lyso-Gb1 decreased from 468 ng/mL (range, 44–1268) to 210 ng/mL (range, 24–893) (*p* < 0.0001), with a large effect size. HGB increased from 123 g/L (range, 54–160) to 131 g/L (range, 60–160), although this did not reach statistical significance (*p* = 0.147). PLT increased from 109 × 10^9^/L (range, 12–511) to 132 × 10^9^/L (range, 11–495) (*p* = 0.019), with a moderate-to-large effect size.

**Figure 2:**
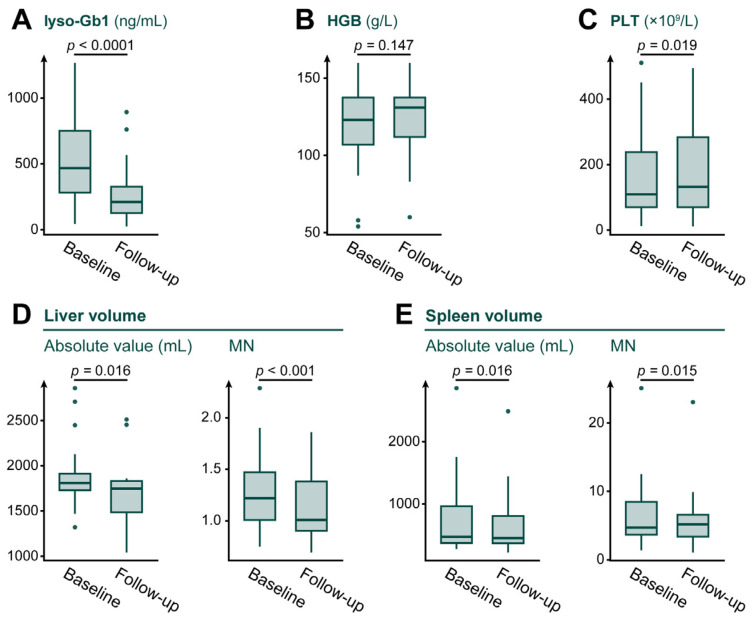
Changes in clinical parameters at baseline and after at least six months of eliglustat treatment. (**A**) lyso-Gb1; (**B**) HGB; (**C**) PLT; (**D**) liver volume; (**E**) spleen volume. Boxplots show the median (center line), interquartile range (IQR; box), and whiskers extending to 1.5× IQR; outliers are plotted as individual dots. Spleen volumes were available for 11 patients. Abbreviations: lyso-Gb1, glucosylsphingosine; HGB, hemoglobin; PLT, platelet count; MN, multiples of normal.

For organ volumes, absolute liver volume decreased from 1808 mL (range, 1321–2861) to 1747 mL (range, 1044–2513) (*p* = 0.016). Liver volume expressed as MN also decreased from 1.22 (range, 0.75–2.29) to 1.01 (range, 0.70–1.86) (*p* < 0.001), with moderate-to-large effect sizes for both measures. Spleen volume analyses were exploratory because eight of the 19 included patients had undergone splenectomy or partial splenectomy, leaving only 11 patients for analysis. Absolute spleen volume decreased from 473 mL (range, 280–2861) to 452 mL (range, 225–2488) (*p* = 0.016), with large effect sizes. Spleen volume expressed as MN was 4.69 (range, 1.37–25.10) at baseline and 5.17 (range, 1.07–23.04) at follow-up; however, a paired analysis indicated an overall reduction (*p* = 0.015) with a large effect size. Figure 2 illustrates changes in clinical parameters, and Table 2 summarizes the baseline and follow-up data in detail.

**Figure 3:**
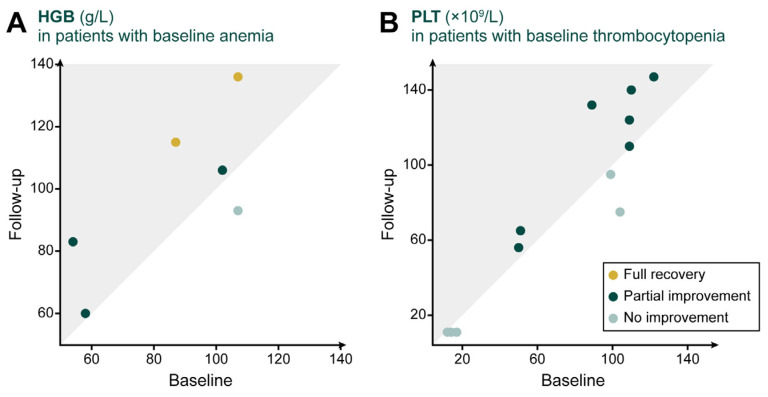
Changes in hematologic parameters at baseline and after at least six months of eliglustat treatment. (**A**) HGB in patients with baseline anemia; (**B**) PLT in patients with baseline thrombocytopenia. Each dot represents one patient. Values within the shaded triangle (above the diagonal line) indicate improvement. Abbreviations: HGB, hemoglobin concentration; PLT, platelet count.

For patients with baseline anemia (defined as HGB < 120 g/L in males or <110 g/L in females) [27] or thrombocytopenia (defined as PLT < 150 × 10^9^/L) [28], changes in hematologic parameters are shown in Figure 3. Among the six patients with anemia, five (83.3%) showed improvement and two (33.3%) achieved full recovery. Among the 12 patients with thrombocytopenia, seven (58.3%) showed improvement.

**Figure 4:**
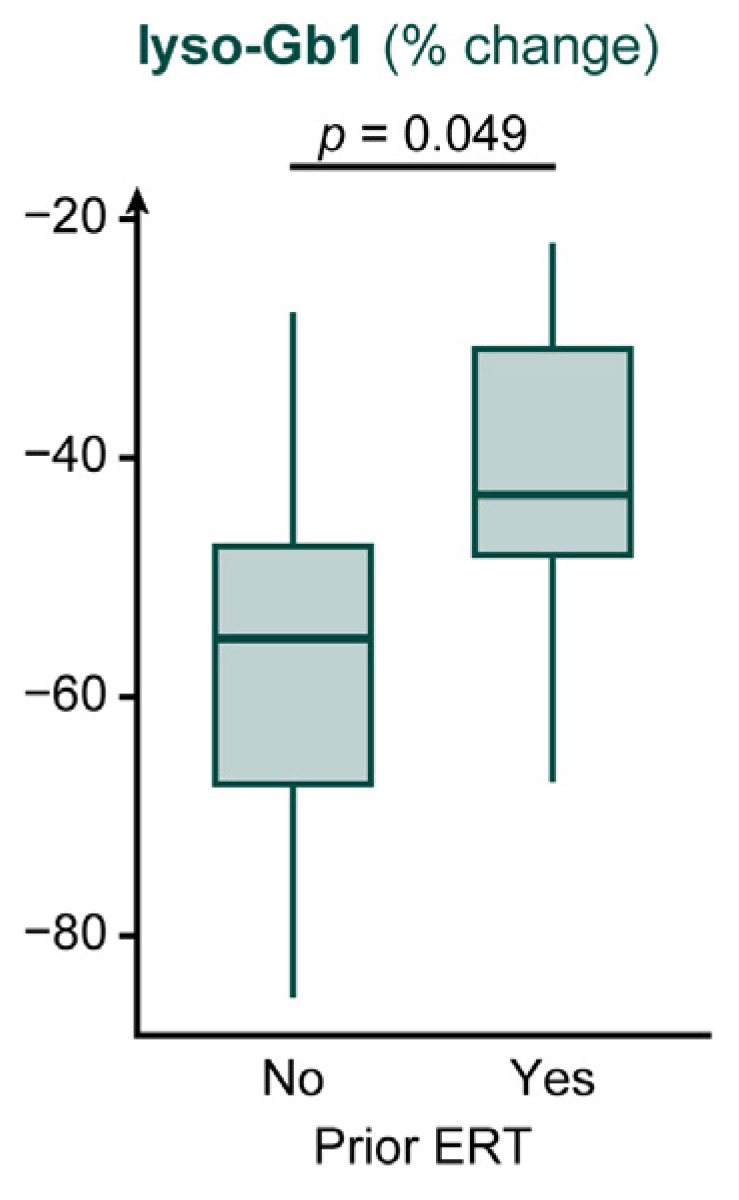
Percentage change in lyso-Gb1 stratified by prior ERT exposure. Boxplots show the median (center line), interquartile range (IQR; box), and whiskers extending to 1.5× IQR; outliers are plotted as individual dots. Abbreviations: lyso-Gb1, glucosylsphingosine; ERT, enzyme replacement therapy.

Subgroup analyses indicated a greater reduction in lyso-Gb1 among patients without prior ERT than among those with prior ERT (−55.1% [−85.2% to −27.8%] vs. −43.1% [−67.1% to −22.0%]; *p* = 0.049), with a large effect size. All subgroup comparisons were exploratory and were not adjusted for multiple testing. In contrast, no significant subgroup differences were observed for HGB or PLT, and stratification by age, sex, disease duration, or splenectomy status did not reveal significant differences. Percentage changes in lyso-Gb1 by prior ERT status are shown in Figure 4, and detailed subgroup results are summarized in Table A1.

### 3.3. Safety Analysis

In the effectiveness analysis group (*n* = 19), five patients (26.3%) reported grade I AEs, including xerostomia (10.5%), abdominal distension (10.5%), nausea (5.3%), constipation (5.3%), and xeroderma (5.3%). One patient (5.3%) reported grade II AEs (xerostomia, gastroesophageal reflux, and arthralgia), which improved after a 50% dose reduction.

In the safety analysis group (*n* = 90), 25 patients (27.8%) reported 60 suspected drug-related AEs, predominantly gastrointestinal events (35 cases, 58.3%) and skin-related events (7 cases, 11.7%). Skin-related AEs consisted of dry skin (42.9%), pruritus (28.6%), urticaria (14.3%), and increased sweating (14.3%). Overall, AEs were transient and self-limited, mostly resolving within 3–5 days without specific interventions. No serious adverse event (SAE) or treatment discontinuation occurred.

## 4. Discussion

This multicenter retrospective study provides early real-world evidence on eliglustat use in Chinese adults with GD1. Overall, eliglustat was associated with rapid substantial reductions in plasma lyso-Gb1, early improvements in hematologic and visceral parameters, and favorable short-term tolerability. These observations complement existing trial and registry evidence and inform settings where long-term infusion-based therapy may be difficult to sustain.

Interpretation should account for the context of GD diagnosis and clinical management in China. Given the progressive nature of GD1, delayed diagnosis and prolonged periods without adequate disease-specific therapy can allow irreversible complications (e.g., avascular necrosis, severe organomegaly, progressive bone disease) to accumulate [29], underscoring the importance of early intervention. In addition, substantial financial barriers, including the high cost of long-term ERT (which may reach several million Renminbi annually), can severely limit access and treatment continuity [13]. As a result, therapy may be initiated at a more advanced stage, including prior splenectomy and marked organ involvement, which can affect both the time course of improvement and the apparent effect size. Comparisons with predominantly Western cohorts are helpful for context, but should be viewed as descriptive rather than inferential, because differences in baseline severity, prior treatment exposure, follow-up duration, and assessment methods across studies can materially shape observed responses.

Against this background, the observed reduction in lyso-Gb1 (median 50.3% [22.0–85.2%]; *p* < 0.0001) falls within the range reported for eliglustat and ERT in prior studies. It is consistent with previously reported outcomes for eliglustat (Cerdelga^®^) in patients without prior treatment (52% reduction over 9 months) [30], and with ERT studies, including imiglucerase in the United States (49% reduction over 3.6 years) [31], and velaglucerase in a multicenter study spanning America, Europe, Africa, and Asia (including India and South Korea) (44.0% at 6 months and up to 57.3% over 3 years) [32]. Collectively, these comparisons support clinically meaningful early substrate control with eliglustat, although longer follow-up is needed to assess durability. This early decline is mechanistically plausible in GD1. By inhibiting GCS and reducing upstream substrate production, eliglustat would be expected to promptly lower lyso-Gb1, supporting its use for short-term monitoring.

Hematologic and visceral manifestations in GD1 reflect multiple mechanisms, including hypersplenism, bone marrow infiltration, and inflammation-related alterations in hematopoiesis [7]. By lowering substrate burden and macrophage activation, eliglustat may attenuate downstream inflammatory signaling, which could plausibly contribute to improvements in HGB and PLT and, over time, reductions in liver and spleen volumes. Although the change in HGB in our study did not reach statistical significance, prior studies provide a useful benchmark. In the phase III eliglustat (Cerdelga^®^) trial, patients without prior treatment achieved a mean increase of 7.6 g/L at 9 months [33], whereas miglustat was associated with only a 0.3 g/L increase after 6 months [34]. Among patients with baseline anemia, the proportions showing improvement (83.3%) and full recovery (33.3%) are clinically notable and are consistent with the possibility of hematopoietic recovery as substrate burden decreases. However, the short follow-up and limited cohort size constrain inference, particularly with respect to statistical significance and between-patient variability.

For PLT, the median increase of 15.7 × 10^9^/L (9.8%; *p* = 0.019) compares favorably with the modest change reported with miglustat over 6 months (3.7 × 10^9^/L) [34], while remaining smaller than long-term gains observed with imiglucerase over 10 years (74.8%) [35], a difference likely attributable, at least in part, to the substantially longer exposure in ERT cohorts. Among patients with baseline thrombocytopenia, the proportion showing improvement (58.3%) is broadly consistent with ERT reports in which approximately half of thrombocytopenic GD1 patients experienced a 21.5% PLT increase after 6 months [36], although cross-study comparisons are constrained by substantial baseline heterogeneity. Notably, for patients starting from extremely low PLT (approximately 20% of normal), normalization can remain difficult even after years of ERT, highlighting a subgroup for whom longer observation and tailored strategies may be particularly important. Consistent with this, the multinational ENCORE study reported PLT improvements after 12 months of eliglustat comparable to ERT, supporting noninferiority [19]. Together, these data underscore the need for adequately powered, head-to-head comparative studies focused on thrombocytopenic patients to better define relative effectiveness and optimize management in this clinically vulnerable population.

Because changes in organ volumes often evolve over longer time horizons, the available follow-up is best interpreted as reflecting early trajectories rather than definitive organ remodeling. Liver volume reductions (−2.9% [−63.5% to 0.2%]) are generally in line with those reported with eliglustat (Cerdelga^®^) (−5% after 9 months) [33] and miglustat (−7% after 6 months) [34], but smaller than reductions reported with ERT, including imiglucerase (−16% to −22% after 6 months) and velaglucerase (−15% after 9 months) [11,37]. Interpretation of spleen-volume changes is more constrained, as only 11 patients were available for analysis. This small and selected subset introduces potential bias and variability and limits robustness. Within these constraints, the observed spleen volume reductions (−15.9% [−19.8% to 12.0%]) are generally in line with prior reports of eliglustat (Cerdelga^®^) (−29% after 9 months) [33] and miglustat (−15% after 6 months) [34]. The high proportion of prior splenectomy (42.1%) further suggests advanced disease at treatment initiation, which may contribute to greater baseline involvement and a slower or more heterogeneous visceral response.

Exploratory subgroup analyses suggested larger reductions in lyso-Gb1 among patients without prior ERT exposure than among those with prior ERT exposure. However, it should be interpreted cautiously, as subgroup sizes were small, the between-group *p* value was borderline, and multiple comparisons increase the risk of chance findings. Overall, these subgroup observations should be regarded as hypothesis-generating rather than causal. Notably, all patients achieved at least a 22.0% reduction in lyso-Gb1, suggesting that clinically meaningful substrate reduction can be observed across patients with varying baseline characteristics and treatment histories, at least over the available follow-up.

The short-term tolerability profile observed in this study appears consistent with long-term safety data for imiglucerase captured in global pharmacovigilance databases [38]. However, retrospective adverse-event ascertainment and remote symptom collection may underestimate event frequency and duration. Longer-term prospective follow-up with standardized safety assessments, particularly with careful attention to potential drug–drug interactions and cardiac risk factors, will be important to refine real-world safety estimates.

Several limitations should be acknowledged. First, the effectiveness analyses included a small number of patients with short follow-up, limiting precision and increasing susceptibility to type II error. Second, the retrospective design introduces selection bias and residual confounding, and the absence of a contemporaneous comparator precludes causal inference. Third, skeletal outcomes were not systematically assessed. Fourth, safety data were collected retrospectively and supplemented by remote, patient-reported symptom updates; therefore, under-ascertainment and misclassification of AEs cannot be excluded, particularly for lower-grade or transient events, despite a central consistency review. Fifth, access-related factors may have influenced both enrollment and baseline severity; in particular, financial barriers that lead to non-initiation or discontinuation of therapy could introduce socioeconomic bias and potentially enrich the analyzed cohort for patients who differ from the broader GD1 population in China with respect to disease severity, healthcare access, or adherence patterns.

## 5. Conclusions

In this multicenter real-world study of Chinese adults with GD1, eliglustat treatment was associated with rapid substantial reductions in plasma lyso-Gb1, early improvements in hematologic and visceral parameters, and favorable short-term tolerability. These findings provide real-world evidence consistent with eliglustat being an effective and well-tolerated oral option for adults with GD1 in routine practice in China. Future studies should prioritize prospective designs with larger cohorts, longer follow-up, standardized assessment of skeletal and quality-of-life outcomes, and more rigorous evaluation of treatment sequencing, adherence, and access-related factors.

## Figures and Tables

**Table 1:** Baseline demographic and clinical characteristics of the effectiveness analysis group (*n* = 19).

Characteristics	Value
Sex	
Male	9 (47.4%)
Female	10 (52.6%)
Age (years)	36 (18 to 55)
Body weight (kg)	60 (42 to 102)
Disease duration (years)	6 (2 to 36)
Metabolizer status	
EM	16 (84.2%)
IM	3 (15.8%)
Prior ERT exposure	8 (42.1%)
Duration (months)	10 (1 to 15)
Splenectomy status	8 (42.1%)
Partial splenectomy	2 (10.5%)
Total splenectomy	6 (31.6%)
lyso-Gb1 (ng/mL)	468 (44 to 1268)
HGB (g/L)	123 (54 to 160)
PLT (×10^9^/L)	109 (12 to 511)
Liver volume	
Absolute value (mL)	1808 (1321 to 2861)
MN	1.22 (0.75 to 2.29)
Spleen volume	
Absolute value (mL)	473 (280 to 2861)
MN	4.69 (1.37 to 25.10)

Categorical variables are presented as frequency (percentage) and continuous variables as median (range). Spleen volumes were available for 11 patients. Abbreviations: EM, extensive metabolizer; IM, intermediate metabolizer; ERT, enzyme replacement therapy; lyso-Gb1, glucosylsphingosine; HGB, hemoglobin; PLT, platelet count; MN, multiples of normal.

**Table 2:** Changes in clinical parameters at baseline and after at least six months of eliglustat treatment.

Parameter	Baseline	Follow-up	% Change	Effect Size (95% CI)	*p* Value
lyso-Gb1 (ng/mL)	468 (44 to 1268)	210 (24 to 893)	−50.3% (−85.2% to −22.0%)	−0.88 (−0.88, −0.88) ^b^	<0.0001
HGB (g/L)	123 (54 to 160)	131 (60 to 160)	3.4% (−15.2% to 53.7%)	0.35 (−0.12, 0.81) ^a^	0.147
PLT (×10^9^/L)	109 (12 to 511)	132 (11 to 495)	9.8% (−35.3% to 48.3%)	0.59 (0.10, 1.07) ^a^	0.019
Liver volume					
Absolute value (mL)	1808 (1321 to 2861)	1747 (1044 to 2513)	−2.9% (−63.5% to 0.2%)	−0.55 (−0.79, −0.16) ^b^	0.016
MN	1.22 (0.75 to 2.29)	1.01 (0.70 to 1.86)	−4.7% (−61.2% to 5.0%)	−0.78 (−0.88, −0.55) ^b^	<0.001
Spleen volume					
Absolute value (mL)	473 (280 to 2861)	452 (225 to 2488)	−15.9% (−19.8% to 12.0%)	−0.87 (−1.56, −0.16) ^a^	0.016
MN	4.69 (1.37 to 25.10)	5.17 (1.07 to 23.04)	−11.0% (−22.7% to 12.1%)	−0.88 (−1.57, −0.16) ^a^	0.015

Categorical variables are presented as frequency (percentage) and continuous variables as median (range). Effect sizes are reported as follows: ^a^ Cohen’s d for the paired *t* test; ^b^ r for the Wilcoxon signed-rank test. Spleen volumes were available for 11 patients. Abbreviations: lyso-Gb1, glucosylsphingosine; HGB, hemoglobin; PLT, platelet count; MN, multiples of normal; CI, confidence interval.

## Data Availability

The data are not publicly available due to personal data protection considerations but are available from the corresponding author upon reasonable request.

## Abbreviations

The following abbreviations are used in this manuscript:

AE	Adverse event
CI	Confidence interval
CTCAE	Common Terminology Criteria for Adverse Events
CYP2D6	Cytochrome P450 2D6
CYP3A	Cytochrome P450 3A
EM	Extensive metabolizer
ERT	Enzyme replacement therapy
*GBA1*	Glucosylceramidase beta 1
GCase	β-glucocerebrosidase
GCS	Glucosylceramide synthase
GD	Gaucher disease
GD1	Gaucher disease type 1
GluCer	Glucosylceramide
HGB	Hemoglobin
IM	Intermediate metabolizer
IQR	Interquartile range
lyso-Gb1	Glucosylsphingosine
MN	Multiples of normal
MRI	Magnetic resonance imaging
PLT	Platelet count
PM	Poor metabolizer
SAE	Serious adverse event
SRT	Substrate reduction therapy
URM	Ultrarapid metabolizer

## Appendix A

**Table A1:** Subgroup analysis of changes in clinical parameters.

Subgroup	lyso-Gb1	HGB	PLT
All patients	−50.3% (−85.2% to −22.0%)	3.4% (−15.2% to 53.7%)	9.8% (−35.3% to 48.3%)
Age (years)	0.38 (−0.54, 1.28) ^a^; 0.417	0.19 (−0.02, 0.61) ^b^; 0.447	−0.07 (−0.97, 0.83) ^a^; 0.873
>36 (*n* = 9)	−46.5% (−67.6% to −27.8%)	6.5% (−6.6% to 53.7%)	3.3% (−21.4% to 27.5%)
≤36 (*n* = 10)	−53.8% (−85.2% to −22.0%)	1.9% (−15.2% to 32.2%)	10.9% (−35.3% to 48.3%)
Sex	−0.30 (−1.20, 0.61) ^a^; 0.525	−0.21 (−1.11, 0.70) ^a^; 0.648	0.20 (−0.70, 1.11) ^a^; 0.648
Male (*n* = 9)	−52.5% (−85.2% to −27.8%)	0.72% (−6.6% to 27.1%)	12.0% (−21.4% to 37.9%)
Female (*n* = 10)	−47.7% (−82.6% to −22.0%)	5.2% (−15.2% to 53.7%)	6.5% (−35.3% to 48.3%)
Disease duration (years)	0.57 (−0.47, 1.60) ^a^; 0.290	−0.84 (−1.87, 0.24) ^a^; 0.131	0.19 (−0.83, 1.20) ^a^; 0.713
>6 (*n* = 9)	−46.5% (−82.6% to −28.4%)	0.0% (−13.1% to 12.4%)	12.0% (−27.9% to 20.5%)
≤6 (*n* = 10)	−60.1% (−85.2% to −31.6%)	5.4% (−15.2% to 53.7%)	9.2% (−35.3% to 37.9%)
Prior ERT exposure	0.96 (−0.02, 1.91) ^a^; 0.049	−0.80 (−1.70, 0.12) ^a^; 0.088	0.36 (−0.56, 1.27) ^a^; 0.453
Yes (*n* = 8)	−43.1% (−67.1% to −22.0%)	1.7% (−13.1% to 7.2%)	11.0% (−27.9% to 48.3%)
No (*n* = 11)	−55.1% (−85.2% to −27.8%)	6.8% (−15.2% to 53.7%)	8.6% (−35.3% to 37.9%)
Splenectomy status	−0.34 (−1.26, 0.58) ^a^; 0.476	−0.78 (−1.68, 0.13) ^a^; 0.094	0.39 (−0.54, 1.30) ^a^; 0.377
Yes (*n* = 8)	−56.6% (−82.8% to −27.8%)	1.83% (−15.2% to 8.8%)	11.0% (−5.3% to 37.9%)
No (*n* = 11)	−45.0% (−85.2% to −22.0%)	6.8% (−13.1% to 53.7%)	8.6% (−35.3% to 48.3%)

Cells without shading report the effect size (95% CI) and *p* value for between-subgroup comparisons. Effect sizes are reported as follows: ^a^ Cohen’s d for the independent-samples *t* test; ^b^ r for the Mann–Whitney U test. Gray-shaded cells report the within-subgroup percentage change from baseline to follow-up, presented as median (range). Abbreviations: lyso-Gb1, glucosylsphingosine; HGB, hemoglobin concentration; PLT, platelet count; ERT, enzyme replacement therapy.

## References

[1] Castillon G., Chang S.C., Moride Y. (2022). Global Incidence and Prevalence of Gaucher Disease: A Targeted Literature Review. J. Clin. Med..

[2] Wang M., Li F., Zhang J., Lu C., Kong W. (2023). Global Epidemiology of Gaucher Disease: An Updated Systematic Review and Meta-analysis. J. Pediatr. Hematol. Oncol..

[3] Lu Y., Gao Q., Ren X., Li J., Yang D., Zhang Z., Han J. (2022). Incidence and prevalence of 121 rare diseases in China: Current status and challenges: 2022 revision. Intractable Rare Dis. Res..

[4] Rossi M., Schaake S., Usnich T., Boehm J., Steffen N., Schell N., Krüger C., Gül-Demirkale T., Bahr N., Kleinz T. (2025). Classification and Genotype-Phenotype Relationships of GBA1 Variants: MDSGene Systematic Review. Mov. Disord..

[5] Camou F., Berger M.G. (2025). Gaucher disease, state of the art and perspectives. J. Intern. Med..

[6] Zhang Z., Yue P., Lu T., Wang Y., Wei Y., Wei X. (2021). Role of lysosomes in physiological activities, diseases, and therapy. J. Hematol. Oncol..

[7] Pandey M.K., Burrow T.A., Rani R., Martin L.J., Witte D., Setchell K.D., McKay M.A., Magnusen A.F., Zhang W., Liou B. (2017). Complement drives glucosylceramide accumulation and tissue inflammation in Gaucher disease. Nature.

[8] Grabowski G.A. (2008). Phenotype, diagnosis, and treatment of Gaucher’s disease. Lancet.

[9] Giuffrida G., Markovic U., Condorelli A., Calafiore V., Nicolosi D., Calagna M., Grasso S., Ragusa M.T.V., Gentile J., Napolitano M. (2023). Glucosylsphingosine (Lyso-Gb1) as a reliable biomarker in Gaucher disease: A narrative review. Orphanet J. Rare Dis..

[10] Carubbi F., Linari S., Spada M. (2026). Glucosylsphingosine (Lyso-Gb1): An Update on Its Use as a Biomarker in Gaucher Disease. Int. J. Mol. Sci..

[11] Barton N.W., Brady R.O., Dambrosia J.M., Di Bisceglie A.M., Doppelt S.H., Hill S.C., Mankin H.J., Murray G.J., Parker R.I., Argoff C.E. (1991). Replacement therapy for inherited enzyme deficiency--macrophage-targeted glucocerebrosidase for Gaucher’s disease. N. Engl. J. Med..

[12] Feng J., Gao Z., Shi Z., Wang Y., Li S. (2023). Patient-reported outcomes in Gaucher’s disease: A systematic review. Orphanet J. Rare Dis..

[13] Qi X., Xu J., Shan L., Li Y., Cui Y., Liu H., Wang K., Gao L., Kang Z., Wu Q. (2021). Economic burden and health related quality of life of ultra-rare Gaucher disease in China. Orphanet J. Rare Dis..

[14] Weinreb N.J., Barranger J.A., Charrow J., Grabowski G.A., Mankin H.J., Mistry P. (2005). Guidance on the use of miglustat for treating patients with type 1 Gaucher disease. Am. J. Hematol..

[15] McEachern K.A., Fung J., Komarnitsky S., Siegel C.S., Chuang W.L., Hutto E., Shayman J.A., Grabowski G.A., Aerts J.M., Cheng S.H. (2007). A specific and potent inhibitor of glucosylceramide synthase for substrate inhibition therapy of Gaucher disease. Mol. Genet. Metab..

[16] Belmatoug N., Di Rocco M., Fraga C., Giraldo P., Hughes D., Lukina E., Maison-Blanche P., Merkel M., Niederau C., Plöckinger U. (2017). Management and monitoring recommendations for the use of eliglustat in adults with type 1 Gaucher disease in Europe. Eur. J. Intern. Med..

[17] Revel-Vilk S., Szer J., Mehta A., Zimran A. (2018). How we manage Gaucher Disease in the era of choices. Br. J. Haematol..

[18] Mistry P.K., Lukina E., Ben Turkia H., Shankar S.P., Baris Feldman H., Ghosn M., Mehta A., Packman S., Lau H., Petakov M. (2021). Clinical outcomes after 4.5 years of eliglustat therapy for Gaucher disease type 1: Phase 3 ENGAGE trial final results. Am. J. Hematol..

[19] Cox T.M., Drelichman G., Cravo R., Balwani M., Burrow T.A., Martins A.M., Lukina E., Rosenbloom B., Ross L., Angell J. (2015). Eliglustat compared with imiglucerase in patients with Gaucher’s disease type 1 stabilised on enzyme replacement therapy: A phase 3, randomised, open-label, non-inferiority trial. Lancet.

[20] Mistry P.K., Balwani M., Charrow J., Kishnani P., Niederau C., Underhill L.H., McClain M.R. (2020). Real-world effectiveness of eliglustat in treatment-naïve and switch patients enrolled in the International Collaborative Gaucher Group Gaucher Registry. Am. J. Hematol..

[21] Lukina E., Watman N., Dragosky M., Lau H., Avila Arreguin E., Rosenbaum H., Zimran A., Foster M.C., Gaemers S.J.M., Peterschmitt M.J. (2019). Outcomes after 8 years of eliglustat therapy for Gaucher disease type 1: Final results from the Phase 2 trial. Am. J. Hematol..

[22] Torralba-Cabeza M., Morado-Arias M., Pijierro-Amador A., Fernández-Canal M.C., Villarrubia-Espinosa J. (2022). Recommendations for oral treatment for adult patients with type 1 Gaucher disease. Rev. Clin. Esp..

[23] Dardis A., Michelakakis H., Rozenfeld P., Fumic K., Wagner J., Pavan E., Fuller M., Revel-Vilk S., Hughes D., Cox T. (2022). Patient centered guidelines for the laboratory diagnosis of Gaucher disease type 1. Orphanet J. Rare Dis..

[24] Bennett L.L., Turcotte K. (2015). Eliglustat tartrate for the treatment of adults with type 1 Gaucher disease. Drug Des. Dev. Ther..

[25] Bracoud L., Ahmad H., Brill-Almon E., Chertkoff R. (2011). Improving the accuracy of MRI spleen and liver volume measurements: A phase III Gaucher disease clinical trial setting as a model. Blood Cells Mol. Dis..

[26] Pastores G.M., Weinreb N.J., Aerts H., Andria G., Cox T.M., Giralt M., Grabowski G.A., Mistry P.K., Tylki-Szymańska A. (2004). Therapeutic goals in the treatment of Gaucher disease. Semin. Hematol..

[27] Liao M.J., Zhang L.S. (2023). Standardized diagnosis and treatment of iron deficiency and iron-deficiency anemia. Zhonghua Nei Ke Za Zhi.

[28] Wang J.X., Zhang F.C., Liu X.Q., Tang C.W., Chen L.A., Han Y. (2020). Expert consensus for diagnosis and treatment of thrombocytopenia in China. Zhonghua Nei Ke Za Zhi.

[29] Platt F.M., d’Azzo A., Davidson B.L., Neufeld E.F., Tifft C.J. (2018). Lysosomal storage diseases. Nat. Rev. Dis. Primers.

[30] Mistry P.K., Lukina E., Ben Turkia H., Shankar S.P., Baris H., Ghosn M., Mehta A., Packman S., Pastores G., Petakov M. (2017). Outcomes after 18 months of eliglustat therapy in treatment-naïve adults with Gaucher disease type 1: The phase 3 ENGAGE trial. Am. J. Hematol..

[31] Murugesan V., Chuang W.L., Liu J., Lischuk A., Kacena K., Lin H., Pastores G.M., Yang R., Keutzer J., Zhang K. (2016). Glucosylsphingosine is a key biomarker of Gaucher disease. Am. J. Hematol..

[32] Elstein D., Mellgard B., Dinh Q., Lan L., Qiu Y., Cozma C., Eichler S., Böttcher T., Zimran A. (2017). Reductions in glucosylsphingosine (lyso-Gb1) in treatment-naïve and previously treated patients receiving velaglucerase alfa for type 1 Gaucher disease: Data from phase 3 clinical trials. Mol. Genet. Metab..

[33] Mistry P.K., Lukina E., Ben Turkia H., Amato D., Baris H., Dasouki M., Ghosn M., Mehta A., Packman S., Pastores G. (2015). Effect of oral eliglustat on splenomegaly in patients with Gaucher disease type 1: The ENGAGE randomized clinical trial. JAMA.

[34] Cox T., Lachmann R., Hollak C., Aerts J., van Weely S., Hrebícek M., Platt F., Butters T., Dwek R., Moyses C. (2000). Novel oral treatment of Gaucher’s disease with N-butyldeoxynojirimycin (OGT 918) to decrease substrate biosynthesis. Lancet.

[35] Weinreb N.J., Goldblatt J., Villalobos J., Charrow J., Cole J.A., Kerstenetzky M., vom Dahl S., Hollak C. (2013). Long-term clinical outcomes in type 1 Gaucher disease following 10 years of imiglucerase treatment. J. Inherit. Metab. Dis..

[36] Grabowski G.A., Barton N.W., Pastores G., Dambrosia J.M., Banerjee T.K., McKee M.A., Parker C., Schiffmann R., Hill S.C., Brady R.O. (1995). Enzyme therapy in type 1 Gaucher disease: Comparative efficacy of mannose-terminated glucocerebrosidase from natural and recombinant sources. Ann. Intern. Med..

[37] Schaison G., Caubel I., Belmatoug N., Billette de Villemeur T., Saudubray J.M. (2002). French results of enzyme replacement therapy in Gaucher’s disease. Bull. Acad. Natl. Med..

[38] Starzyk K., Richards S., Yee J., Smith S.E., Kingma W. (2007). The long-term international safety experience of imiglucerase therapy for Gaucher disease. Mol. Genet. Metab..

